# The role of weight stigma in weight regain in bariatric surgery

**DOI:** 10.3389/fendo.2022.1076696

**Published:** 2022-12-06

**Authors:** Mary S. Himmelstein, Kristen A. Knepp, Sean M. Phelan

**Affiliations:** ^1^ Department of Psychological Sciences, Kent State University, Kent, OH, United States; ^2^ Department of Family and Community Medicine, Northeast Ohio Medical University, Rootstown, OH, United States; ^3^ Division of Health Care Delivery Research, Robert D. & Patricia E. Kern Center for the Science of Healthcare Delivery, & Mayo Clinic Alix School of Medicine, Mayo Clinic, Rochester, MN, United States

**Keywords:** bariatric surgery, weight regain, weight stigma, weight bias internalization, psychological distress

## Abstract

Obesity is highly stigmatized, and individuals who undergo bariatric surgery are subject not only to weight stigma, but also to stigma related to the procedure itself. Patients lost to follow-up after surgery make estimating the amount of regain occurring after surgery difficult, and often patients fail to follow up due the shame of weight regain. Patients report difficulty following the diet necessary to maintain weight loss. Additionally, when they seek support after surgery, they often encounter stigmatizing messaging related to weight. Weight bias internalization, weight stigma, and stigma about having the surgery all contribute to feelings of social isolation, disordered eating, and reduced motivation to engage in physical activity. In this chapter, we present evidence for the impact of stigma on bariatric surgery outcomes and discuss the behavioral, physiological, and emotional processes that contribute to weight regain.

## Introduction

The prevalence of obesity within the United States increased to 41% in 2020 (1). People with obesity – a highly stigmatized condition – experience high rates of weight stigma or stereotyping, mistreatment, and social devaluation related to body weight (2). These experiences occur across many domains (e.g., media, employment, retail settings, education, interpersonal relationships, exercise settings), but are rampant in healthcare (2, 3). Physicians, other health care providers, and medical trainees (4–6) have all endorsed bias against individuals with high weight, including providers specializing in obesity medicine (7). Over 60% of patients with overweight and obesity report prior stigma from doctors (3, 8, 9).

In the wake of a growing body of evidence demonstrating that weight stigma is detrimental to physical and psychological health (10, 11), multiple medical professional organizations have put out calls to end weight stigma in practice (12–15). While research on weight stigma in the general population has exploded over the last two decades, comparatively less is known about weight stigma in bariatric patients. People who undergo surgical intervention to lose weight experience weight stigma that is compounded by stigma related to having the procedure (16–18), which can cause stress and contribute to weight regain through several causal pathways (19). In this article, we will present evidence for the impact of stigma on bariatric surgery outcomes and discuss the behavioral, physiological, and emotional processes that contribute to weight regain.

About half of Americans have tried to lose weight in the last year (20). Definitions of what constitutes successful weight loss have changed in weight loss studies over the years (e.g., 10% of body weight, 5% of body weight), as have definitions for weight regain (21). In the behavioral weight loss literature, success is typically defined as 5 to 10% of body weight loss (22). Follow-up studies, however, indicate that approximately one-third of patients regain any weight lost within the first 12 months and nearly all individuals regain all of the weight lost within five years (22).

Successful weight loss outcomes appear better in the bariatric surgery literature. At five-year follow-ups with patients who underwent different types of bariatric surgery, weight losses of 16% to 23% total body weight loss were generally maintained (23, 24). After 15 years, patients maintained weight losses equal to about half of the initial weight lost in the first year after surgery (25). However, meta-analyses and individual studies of long-term weight loss maintenance note that prevalence of weight loss maintenance after bariatric surgery is hard to parse because very few studies include long-term follow up (26).

Follow-up with patients after bariatric surgery is generally poor (27). Thus, estimates of weight loss maintenance are necessarily biased as they are based on available data from patients who were not lost at follow-up (24, 26), and weight loss is typically greater among patients who attend follow-up appointments (28). The conclusion typically drawn from data on follow-up care is to encourage patients to attend follow-up appointments, but this conclusion fails to acknowledge that a central reason for failure to attend follow-up visits is healthcare avoidance due to shame from weight regain (29, 30). Indeed, patients are very likely to miss follow-up appointments when weight regain occurs (31). Patients often blame themselves for failure to attend follow-ups after surgery in both medical appointments and support groups (32), both of which tend to emphasize personal responsibility in dietary adherence, which serves to reinforce stigma and shame (32).

As in the behavioral weight loss literature, one of the major reasons for weight regain is difficulty with adherence to strict dietary guidelines after surgery (31, 33). These guidelines typically recommend soft foods for the first six months, followed by limiting dietary intake to 1200 to 1500 calories per day for women and 1500 to 1800 calories for men, while severely limiting or abstaining from high fat or high sugar foods (22, 34). It is noteworthy to mention that these guidelines are similar to those used during the semi-starvation protocol of the Minnesota study on the Biology of Human Starvation (calorie limit of 1570 per day).

While providers typically view the difficulty of maintaining the bariatric diet as simply a part of the commitment to living a healthy lifestyle (and non-adherence as a lack of commitment to lifestyle changes or willpower, reinforcing stigma), patients report that adherence is complicated by social, work, and family demands; they cite the difficulty of socializing with friends while maintaining this rigid diet, problems finding ways to maintain the diet in food environments not conducive to calorie restriction or healthy eating, and struggles with hunger (31, 33, 34). Patients often blame themselves, describing the weight regain as a lonely struggle in which they feel largely unsupported by health professionals (29). Many patients report wanting proactive support from their providers, access to dieticians, access to physiotherapists, and access to psychologists (29).

Patients view the factors noted above, as well as support from peers, as the most important contributors to helping them to maintain weight reductions, especially in their first year post-surgery. Patients simultaneously felt that support in these areas was infrequent or inadequately provided (35). Patients also blame themselves for problems that occur as the result of the surgery, which impede their ability to maintain physical activity (e.g., chronic pain, lack of energy). They view these problems as their fault given the elective nature of bariatric surgery (30).

This self-blame for inability to adhere to diet, failure to attend medical appointments, and physical effects of the surgery all likely contribute to or exacerbate existing weight bias internalization. Weight bias internalization encompasses self-stereotyping and self-devaluation based on body weight (36). Experiences with weight stigma (2) and weight bias internalization (37) are known to be associated with avoidance of healthcare (3) and disordered eating, including binge eating (38). Qualitative evidence suggests that post-bariatric surgery patients do not recognize these links (34). Instead, patients tend to blame disordered eating, failure to adhere to the rigid dietary standards, and the resultant weight regain on themselves, reifying weight bias internalization or self-stereotyping and devaluation related to body weight (34).

Weight bias internalization and experiences with weight stigma elicit both behavioral reactions to stress associated with weight gain (e.g., overeating (2, 37); and physiological reactions to stress (e.g., elevated stress hormones (39–41). Stress hormones contribute to weight gain by encouraging accumulation of abdominal adiposity, and cortisol drives both hunger and eating behavior (42). Experiencing weight stigma has been shown to predict 24-hour elevated cortisol (10, 43) and can cause acute elevations in cortisol in experimental work (40, 41). Experiencing the stigma associated with obesity stereotypes relevant to bariatric surgery likely has the same effect. Weight bias internalization is associated with blunted cortisol reactivity, suggesting it may serve as a chronic rather than acute stressor (39).

Of concern for bariatric surgery patients, weight bias internalization is strongly tied to higher body weight, reduced motivation to exercise, and disordered eating (37). In the behavioral weight loss literature, changes of 5 to 10% in body weight after weight loss do not predict changes in weight bias internalization, even when weight bias internalization is directly targeted in treatment (44). That is, weight bias internalization appears particularly resistant to change even in the face of substantial weight loss and when addressed in patients’ therapy. Since weight bias internalization is associated with body weight, individuals who undergo bariatric surgery at lower BMIs may also have lower levels of bias internalization, and thus may be less affected by its influence on stress. In addition, weight bias internalization has been shown to be prevalent across the lifespan, including in adolescents (45) and even very young children (46), underscoring its pervasiveness in society. Although, experiences with weight stigma are associated with higher weight bias internalization scores, there are also individuals who score high in weight bias internalization in the absence of an acute experience with weight stigma (45). Such evidence suggests that even those who undergo surgery at younger ages, prior to their exposure to weight bias in adult contexts and settings, may already be dealing with internalization and its impact on stress and well-being.

Studies on the ability of pre-bariatric surgery weight bias internalization scores to predict weight loss are mixed, with multiple studies finding no predictive effects of weight bias internalization on weight loss (34, 47) and others demonstrating a negative relationship between weight bias internalization and weight loss (48, 49). Pre-surgery weight bias internalization is strongly tied to depression, anxiety, and lower quality of life independent of BMI (50). It also appears to be a strong risk factor for many patterns of disordered eating. Multiple studies show that pre-surgery weight bias internalization scores predict disordered eating after surgery (e.g., binge eating, food restriction, laxative use, disinhibited eating (34, 51–53).

Weight bias internalization also predicts emotional eating, turning to food during times of stress, less adherence to dietary programs, and excessive thoughts about food after bariatric surgery (34, 51, 54, 55). Compounding disordered eating, weight bias internalization before surgery also predicts lower levels of physical activity and self-efficacy for exercise after surgery (56). Moreover, weight bias internalization before surgery predicts failure to attend follow-up appointments after surgery (57), meaning that patients most at risk for regain related to diet and physical activity may avoid follow-up care.

The relationships among experiences with weight stigma and eating as well as weight loss after bariatric surgery are less well studied. Like weight bias internalization, experiences with weight stigma after bariatric surgery are similarly tied to disordered eating, including binge eating and food restriction (34, 51, 58, 59). Among bariatric surgery candidates, frequency of stigmatizing experiences within the past month is associated with depression and anxiety, self-esteem, and body image distress (59). As in weight bias internalization, weight stigma is similarly tied to lower motivation to exercise after surgery (56). Patients typically expect to experience weight stigma as they begin to regain weight after surgery and are likely to limit their social activities and avoid medical care (29, 51). Weight stigmatizing messaging is reinforced in social support groups for bariatric patients and often reinforced by medical professionals when patients have trouble adhering to the dietary plan (32, 34).

One factor contributing to experiences with weight stigma and weight bias internalization is the belief that obesity is caused by one’s lack of willpower, laziness, and poor choices (60), despite evidence that obesity has multiple complex social, genetic, and physiological contributors (61). This is consistent with attribution theory, which asserts that people feel less empathy for and are less willing to help a person with a condition that is perceived to have been self-inflicted, as compared to a condition that is perceived to have an external cause (60).

These attitudes underscored by attribution theory extend to stigma surrounding obesity treatments with proven efficacy like bariatric and metabolic surgery or weight loss pharmaceutical therapy. These treatments for obesity are viewed by many to be a “shortcut” or “the easy way” to lose weight, and comments to this effect are very stigmatizing (17, 18, 62–64). Bariatric surgery is also perceived as a cosmetic procedure, solely for physical appearance rather than health reasons (65). Causal evidence from experimental studies suggests that when research participants are shown a photo of an individual and are told she lost weight though surgery, they rate her as more lazy, more sloppy, less competent, less sociable, less employable, and less attractive than an individual who lost weight nonsurgically *via* dietary changes (66, 67). A second study replicated these results, demonstrating the differences in how the woman was perceived were explained by perceptions that she was personally responsible for her weight loss. That is, participants believed that individuals who had surgery should not “receive credit” for weight lost in this manner (68). Indeed, as many as 87% of bariatric surgery patients report hearing negative comments about surgery as a means to lose weight (17).

Many patients expect to experience less stigma after bariatric surgery, particularly from providers, but studies show it does not manifest that way (34, 69). This may be explained *via* lingering stigma from perceptions about bariatric surgery. Thus, the stereotype of people with obesity as lazy and lacking willpower persists and can be reinforced by choosing a treatment with proven efficacy. Indeed, the procedure of bariatric surgery itself is significantly underutilized (70). While the reasons for underutilization are multifaceted, weight stigma and poor physician-patient communication are likely partly responsible; weight stigma and stigma from the procedure are both likely significant barriers to bariatric surgery (70).The impact of these biases on people who have received bariatric surgery may be considerable. People who feel they are at risk of confirming a group stereotype or fear they will be seen in terms of group stereotypes experience stereotype threat, a reaction characterized by elevated anxiety and a stress response (10, 43).

While the question of how to address the impact of stigma, weight bias internalization, and stress on weight regain is unanswered, evidence from basic psychology research points to potential strategies that should be evaluated in this applied setting. For example, discussing with patients how to identify the effects of stigma and stereotype threat, with the understanding that such factors are the source of their stress and anxiety, may help patients be aware of the situations that trigger threat for them. In this way, patients can understand that the anxiety and negative emotions have an external cause, and one that they can try to avoid (71). Ongoing post-surgical mental health care is rare; however, patients may benefit from the opportunity to work through the difficult and confusing emotions, feelings of self-doubt, and fear of failure that can occur. A mental health professional could help them reframe difficult feelings and identify barriers outside of themselves that affect weight loss, so that setbacks are not internalized as signs of personal failure. Some providers may also fail to inform patients that there are medications and revision procedures that can help address weight regain, out of fear that patients will not try as hard to lose weight. Instead, counseling patients that weight loss will continue to be difficult after surgery and that there are strategies to help them could normalize challenges following surgery and prevent patients from experiencing the shame associated with weight regain, which may prevent them from seeking follow-up care.

## Conclusion and future directions

In summary, we have provided evidence that weight bias is less well-studied, and thus less understood, in the bariatric population, as compared to the general population. Patients who have undergone weight loss or metabolic surgery experience self-stigma, in the form of internalized weight bias, as well as stigmatizing messages from other people in their lives, including medical providers. Currently, our understanding of weight bias in bariatric surgery candidates is limited by the lack of research to date on how weight stigma manifests in this rapidly increasing group of patients, as well as the consequences of such stigma. If, as a medical community, the goal is to optimize weight and health outcomes for bariatric patients post-surgery, we must allocate time and effort to understanding why these individuals are not following up with providers and how various types of stigma affect surgical success. As noted in this chapter, behavioral reactions to stress and bias internalization can work separately or together to make weight loss more difficult. Medical providers, particularly obesity medicine physicians working with bariatric patients, need to recognize and comprehend the multiple barriers patients face at each stage of the surgical process when providing evaluation and follow-up care.

Further, a number of questions remain about patient care following bariatric surgery, and how the decision to seek follow-up care may be impacted by weight bias. First, does this *expectation* of less stigmatizing healthcare encounters translate to greater adherence to follow-up care among individuals who undergo bariatric surgery? Evidence presented in this chapter and elsewhere suggests that patients avoid healthcare appointments as a result of shame and self-blame due to weight regain (29, 30), and patients are more likely to miss follow-up appointments under these circumstances (31).

It follows that weight bias internalization on the part of the patient contributes to less frequent encounters with providers postoperatively, and follow-up medical care that may be considered inconsistent at best. In turn, lack of appropriate follow-up care after weight loss surgery may contribute to suboptimal outcomes in terms of patients’ amount of weight loss, as well as general health and wellness. Societal messaging, as well as messaging within the medical community, suggests that individuals who lose weight *via* bariatric surgery are “taking the easy way out” of their weight and health struggles. Moreover, these patients are viewed more negatively than individuals who might attempt weight management by way of sweeping and potentially unsustainable lifestyle changes that do not involve surgery (e.g., extreme deprivation and dietary restriction, meticulous food and activity tracking, intense exercise regimens, and relentless “hard work” in general). Such messages are unhelpful, and even harmful, in efforts to reduce stigma and bias for individuals struggling with overweight and obesity, and in particular, bariatric patients.

Additionally, if postsurgical patients continue to feel stigmatized by obesity medicine providers – contrary to their initial expectations – does this lead to poorer postoperative consequences, suboptimal health behaviors, and ultimately greater avoidance of healthcare long-term? Might bariatric surgery patients delay seeking treatment due to a self-perception as a “failure” for achieving less weight loss than desired following the procedure? Are postsurgical patients lost to follow-up, in part due to concerns that their medical providers will be disappointed in their progress (or lack thereof)? Future research must be dedicated to answering these complex, yet critically important questions to improve healthcare experiences and outcomes for individuals with obesity, including bariatric surgery candidates.

## Author contributions

All authors meet the requirements for authorship. MH led writing of the manuscript, KK and SP made substantial and important contributions. All authors contributed to the article and approved the submitted version.
